# Molecular-Based Detection of *Enterocytozoon bieneusi* in Farmed Masked Palm Civets (*Paguma larvata*) in Hainan, China: A High-Prevalence, Specificity, and Zoonotic Potential of ITS Genotypes

**DOI:** 10.3389/fvets.2021.714249

**Published:** 2021-10-01

**Authors:** Wei Zhao, Guang-Xu Ren, Yu Qiang, Jiaqi Li, Jinkang Pu, Yun Zhang, Feng Tan, Huicong Huang, Shaohui Liang, Gang Lu

**Affiliations:** ^1^Key Laboratory of Tropical Translational Medicine of Ministry of Education, Hainan Medical University, Haikou, China; ^2^Department of Parasitology, Wenzhou Medical University, Wenzhou, China; ^3^Key Laboratory of Tropical Translational Medicine of Ministry of Education, National Health Commission (NHC) Key Laboratory of Control of Tropical Diseases, Hainan Medical University, Haikou, China; ^4^Hainan Medical University-The University of Hong Kong Joint Laboratory of Tropical Infectious Diseases, Hainan Medical University, Haikou, China; ^5^Department of Pathogenic Biology, Hainan Medical University, Haikou, China; ^6^Academician Workstation of Hainan Province, Hainan Medical University, Haikou, China

**Keywords:** *Enterocytozoon bieneusi*, masked palm civets, molecular-based detection, zoonotic, Hainan

## Abstract

*Enterocytozoon bieneusi* is a microsporidian and zoonotic species. This study investigated the prevalence and distribution of *E. bieneusi* genotypes in farmed masked palm civets using nested PCR, as well as assessed their zoonotic potential by phylogenetic analysis of the ITS region of the rRNA region. Here, we collected 251 fecal specimens from farmed masked palm civets (*Paguma larvata*) from the Hainan Island, China. In total, 128 of 251 samples were positive for *E. bieneusi*, with an average infection rate of 51.0%. Seventeen genotypes were identified including 12 known genotypes—HNR-VI (*n* = 56), SHR1 (*n* = 45), SHW7 (*n* = 6), KIN-1 (*n* = 3), D (*n* = 3), New1 (*n* = 3), EbpC (*n* = 2), CHC5 (*n* = 1), CHG19 (*n* = 1), CHN4 (*n* = 1), EbpA (*n* = 1), and Henan-III (*n* = 1)—and five novel genotypes (HNPL-I to HNPL-II; one each). Phylogenetic analysis categorized these genotypes into two groups. Thirteen of them were members of the zoonotic group 1, and the remaining four genotypes were in group 12. This study has shown that the infection rates of *E. bieneusi* in masked palm civets from Hainan were relatively high and provide baseline data to control and prevent microsporidiosis in farm-related communities. Therefore, infections in masked palm civets with zoonotic genotypes D, EbpC, CHN4, EbpA, KIN-1, and Henan-III should be considered potential threats to public health.

## Introduction

*Enterocytozoon bieneusi* (microsporidia), a microorganism, infects the intestinal epithelial cells of humans (1). It is prevalent throughout the world and has been found in various animals including mammals, birds, and reptiles (2). Also, the infectious spores of *E. bieneusi* are abundant in the environment, including ditches and other water surfaces, indicating the possibility of waterborne disease (3). Therefore, there could be variable transmission modes, such as fecal–oral, oral–oral route, or *via* intake of infected food or water (4).

The sequencing of a single ITS region of the rRNA gene has identified over 600 genotypes of *E. bieneusi* (5). Elsewhere, studies have shown that there seems to be a close relationship between *E. bieneusi* genotypes with those of humans and animals (49 genotypes are found in both animals and humans) (6, 7). Meanwhile, *E. bieneusi* exhibits high genetic diversity within the host species and environmental sources (6). The identified genotypes were sorted into 13 clades with groups 1 and 2 having zoonotic potential, and the remaining groups had host-specific/wastewater genotypes (2, 8). Thus, the epidemiological surveys need to explore the genotyping of *E. bieneusi* isolates with possible zoonotic potential to comprehend the epidemiology of human microsporidiosis.

Masked palm civets (*Paguma larvata*) are wild mammals domesticated as new farm animals in southern China and have become the most abundant in human-inhabited environments. More than 100 farms are currently raising civets in Hainan, China, due to suitable climate and an ideal land compared with the mainland. Some studies have suggested that they could transmit some zoonotic pathogens, including bacteria, viruses, and parasites (9–11). Until now, only a single study has studied *E. bieneusi* effects in masked palm civets (12). Therefore, here, we aimed to estimate the prevalence and identify varied genotypes of *E. bieneusi* in the civets farmed in Hainan, China, through PCR of the ITS region in the rRNA gene. Moreover, our study has assessed the potential zoonotic transmission of *E. bieneusi* isolates using neighbor joining (NJ) method-based phylogenetic analysis.

## Materials and Methods

### Ethics Statement

The experimental protocol was sanctioned by the Research Ethics and Animal Ethics Committee of the Hainan Medical University. During the entire procedure, no animals were injured.

### Specimen Collection

**Figure 1:**
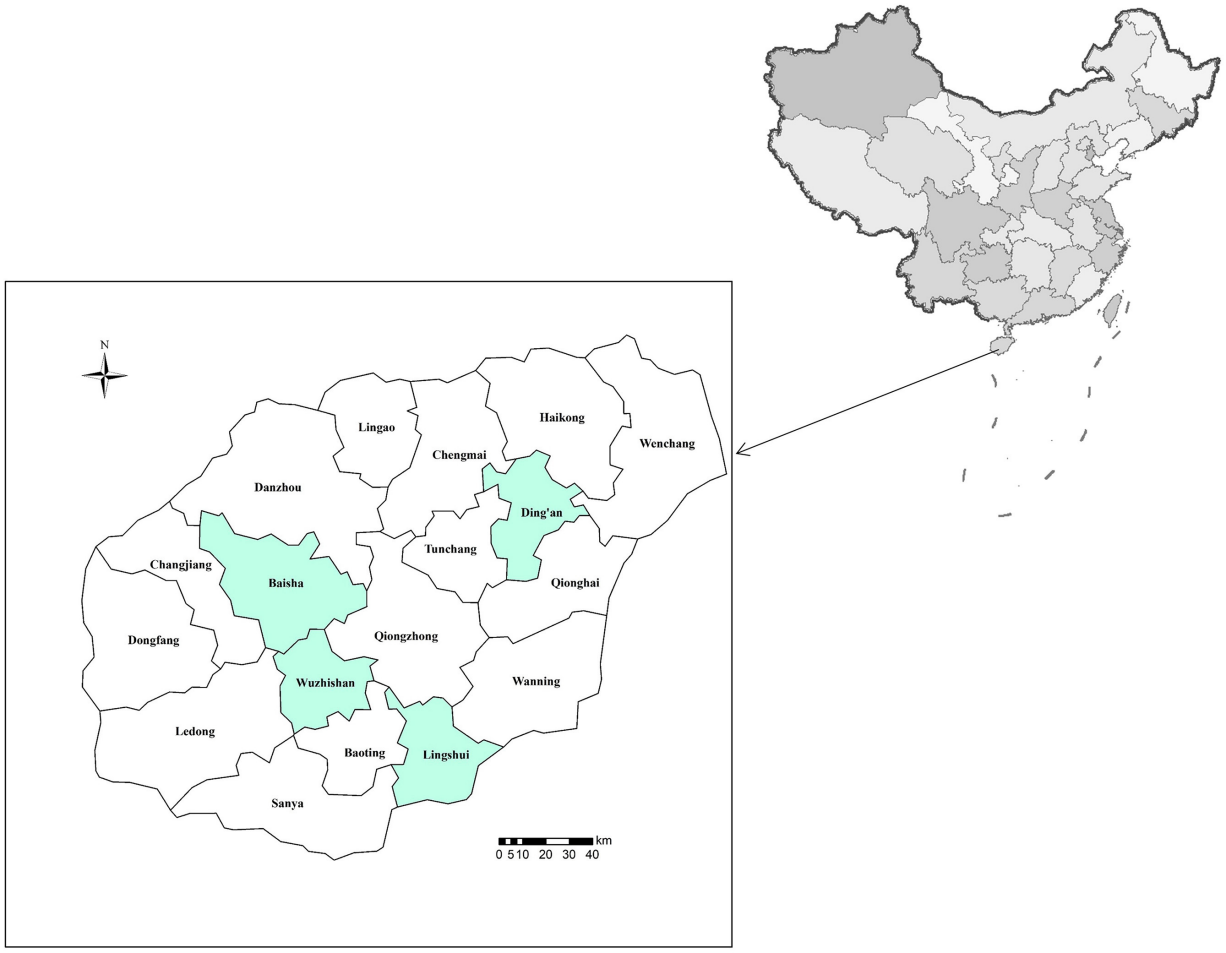
The colored locations where samples were collected.

Between May 2019 and March 2021, ~10 g of fecal specimens was collected from 251 masked palm civets farmed in five farms from four cities of Hainan (the southernmost province of China) (Figure 1). Among them, 72 from Baisha (farm 1), 108 from Ding'an (50 in farm 2 and 58 in farm 3), 56 were from Lingshui (farm 4), and 15 from Wuzhishan (farm 5) (Table 1). Only those farms were chosen whose owners agreed to participate in the study and had easy access to animals for sampling. The masked palm civets were categorized into groups of two (a male and a female) per cage. The fecal samples were collected immediately after excretion and kept in separate plastic bags. Notably, only one specimen was collected per cage to avoid duplicate sampling. All fecal samples were stored at 4°C. The collected samples accounted for ~30% of the total animals on each farm. All animals were aged 2–4 years and were healthy.

**Table 1:** Prevalence and genotype distribution of *Enterocytozoon bieneusi* isolates in civets in Hainan Province.

**Locations**	**Farms**	**Positive/examined (%)**	**Genotype/s (*n*)**
Baisha	Farm 1	47/72 (65.3)	SHR1 (45), HNPL-IV (1), HNPL-V (1)
Ding'an	Farm 2	24/50 (48.0)	HNR-VI (15), Macaque4 (2), HNPL-I (1), HNPL-II (1), SHW7 (3), D (1), CHC5 (1)
Ding'an	Farm 3	43/58 (74.1)	HNR-VI (36), SHW7 (3), CHN4 (1), KIN-1 (2), Henan-III (1)
Lingshui	Farm 4	2/56 (3.6)	EbpA (1), EbpC (1)
Wuzhishan	Farm 5	12/15 (80.0)	HNR-VI (5), D (2), CHG19 (1),EbpC (1), KIN-1 (1), HNPL-III (1), Henan-III (1)
Total	128/251 (50.2)	HNR-VI (56), SHR1 (45), SHW7 (6), KIN-1 (3), D (3), Macaque4 (2), EbpC (2), Henan-III (2), CHC5 (1), CHG19 (1), CHN4 (1), EbpA (1), HNPL-I (1), HNPL-II (1), HNPL-III (1), HNPL-IV (1), HNPL-V (1)	

### DNA Extraction

All the fecal specimens were sieved through a 45-μm mesh, followed by centrifugation (1,500 g; 10 min). We used 1,000 μl of InhibitEX® buffer solution to homogenize each processed sample (200 mg). Next, the QIAamp DNA stool mini kit was used to isolate DNA from the homogenized samples. The procedures and utilized reagents were provided by the manufacturer, but to obtain high yield of DNA, the lysis temperature was increased to 95°C instead of 70°C. The extracted DNA was stored at −20°C.

### PCR Amplification

We amplified 390 bp of the rRNA gene, containing SSU rRNA gene (3′ end; 76 bp), ITS region (243 bp), and LSU rRNA gene (5′ end; 70 bp) to analyze DNA for *E. bieneusi*, and the primers (EBITS3/EBITS4 and EBITS1/EBITS2.4) and the PCR parameters (the two sets of cycling parameters were 35 cycles of 94°C for 30 s, 57°C for 30 s, and 72°C for 40 s and 30 cycles of 94°C for 30 s, 55°C for 30 s, and 72 C for 40 s, with both of them having a final extension step at 72°C for 10 min) were designed by Buckholt et al. (13). The PCR amplification was done using the TaKaRa (Dalian, China) Taq DNA Polymerase with no template DNA as the negative control to test minor contamination. All PCR products were electrophoresed using a 1.5% agarose gel, followed by GelRed staining.

### Nucleotide Sequencing and Analysis

We adopted the primers used for secondary PCR to sequence the positive secondary PCR products for *E. bieneusi*. First, an ABI PRISM 3730 XL DNA Analyzer (Thermo Fisher Scientific, Waltham, MA, USA) with the SinoGenoMax Biotechnology Co., Ltd. (Beijing, China) and the Big Dye Terminator v3.1 Cycle Sequencing Kit were used to purify the sequences. Sequence accuracy was verified through bidirectional sequencing. Nucleotide sequences from this study were aligned together; reference sequences were obtained from the GenBank database using the Clustal X 1.83 (http://www.clustal.org/). In our study, first published names were given to the obtained *E. bieneusi* genotypes if they were identical to the GenBank sequences (14). DNA sequencing was done to verify the genotypes that generated ITS sequences with single nucleotide substitutions/deletions/insertions. Here, the novel genotypes were labeled by adding roman numbers after HNPL (Hainan *P. larvata*). Finally, in this study, we named the genotypes based on the 243 bp of the *E. bieneusi* ITS gene region (14).

### Phylogenetic Analysis

The Mega7 (http://www.megasoftware.net/) program was used to build a NJ tree to assess the genetic relationship between the obtained *E. bieneusi* novel ITS genotypes. A comparative analysis was performed with the known genotypes using the Kimura-2 parameter model. The bootstrap analysis method with 1,000 replicates was used to assess the reliability of these trees.

### Nucleotide Sequence Accession Numbers

The GenBank database accession numbers of the representative nucleotide sequences were MZ229903–MZ229907.

### Statistical Analysis

Data entry and analysis were performed using Statistical Package for the Social Sciences (SPSS) 19.0 software. We used the χ^2^-test to compare *E. bieneusi* prevalence among different farms. *p* < 0.05 was the threshold of statistical significance.

## Results

### Infection Rates of *Enterocytozoon bieneusi* in Masked Palm Civets

One hundred twenty-eight of 251 specimens from masked palm civets amplified the ITS region of the rRNA gene. Hence, they were *E. bieneusi* positive and had an average infection rate of 51.0%. The occurrence rate of the *E. bieneusi* in these animals from the five farms showed a significant difference (χ^2^ = 73.94; *p* < 0.001). In particular, the animals from farm 5 demonstrated the highest prevalence rate of *E. bieneusi* (12/15, 80.0%), followed by farm 3 (43/58, 74.1%), farm 1 (47/72, 65.3%), farm 2 (24/50, 48.0%), and farm 4 (2/56, 3.6%) (Table 1). They differ by pairwise comparisons, as follows: farm 1 vs. farm 4 (χ^2^ = 50.7; *p* < 0.001); farm 2 vs. farm 3, farm 4, and farm 5 (χ^2^ = 7.8; *p* = 0.005; χ^2^ = 28.2; *p* < 0.001; χ^2^ = 4.9; *p* = 0.029, respectively); farm 3 vs. farm 4 (χ^2^ = 59.4; *p* < 0.001); and farm 4 vs. farm 5 (χ^2^ = 43.7; *p* < 0.001).

### Genotype Distribution of *Enterocytozoon bieneusi*

Sequencing and multiple sequence alignment identified 17 genotypes in the masked palm civets. They comprised 12 known genotypes (CHC5, CHG19, CHN4, D, EbpA, EbpC, Henan-II, HNR-VI, KIN-1, macaque4, SHR1, and SHW7) and five novel genotypes (HNPL-I to HNPL-V; MZ229903 to MZ229907). Among them, the HNR-VI genotype was 43.8% (56/128) of all the *E. bieneusi* isolates and was followed by SHR1 (35.2%; 45/128), SHW7 (4.7%; 6/128), and both genotypes D and KIN-1 were 2.3% (3/128) each. In contrast, the other genotypes occurred at a lower frequency: 1.6% (2/128) for genotypes EbpC, Henan-III, and macaque4 each, and 0.8% (1/128) for genotypes CHC5, CHG19, CHN4, EbpA, and HNPL-I to HNPL-V each. Subsequently, the two most dominant genotypes (HNR-VI and SHR1) were distributed in different regions. Specifically, genotype HNR-VI was detected in Ding'an and Wuzhishan, whereas genotype SHR1 was identified in Baisha (Table 1).

### Genetic Relationships of ITS Genotypes

Both HNPL-I (MZ229903) and HNPL-II (MZ229904) novel genotypes had one single-nucleotide polymorphism (SNP), whereas the EbpC (AF076042) genotype had one at nucleotide sites 56 (G → A) and 98 (G → A) of the ITS region. The similarity between HNPL-III (MZ229905) and EbpD (MG736664) genotypes was 99.18%, with two base differences at nucleotide sites 23 (C → T) and 241 (C → T), respectively. There were two base differences between HNPL-IV (MZ229906) and HNPL-V (MZ229907) genotypes, and when compared with the SHR1 (MN523336) genotype, they both had a base variation. The former had a difference at site 83 (G → A) and the latter at site 195 (G → A).

**Figure 2:**
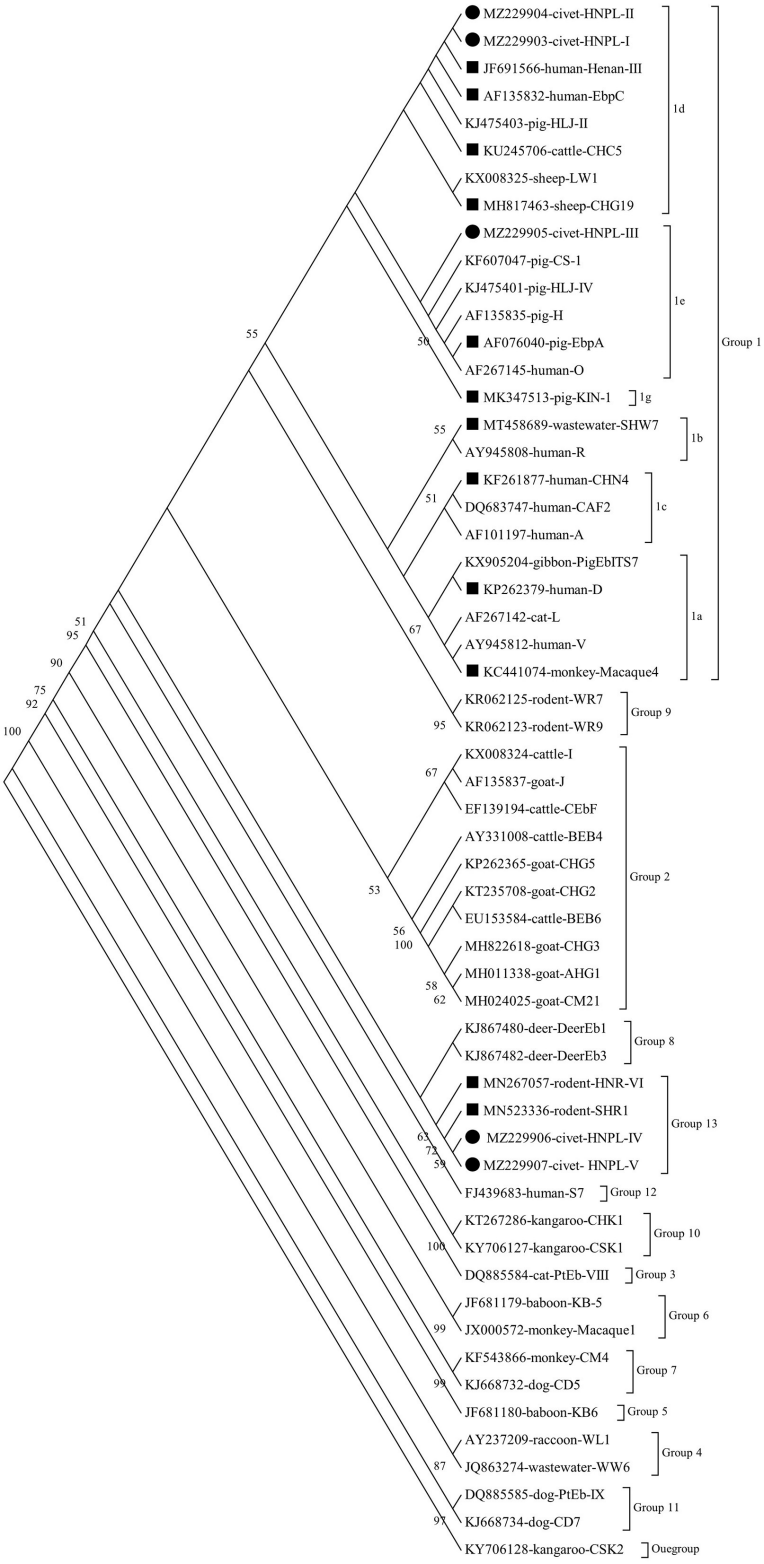
Phylogenetic relationship of the *Enterocytozoon bieneusi* genotypes. A neighbor joining (NJ) tree was done to infer the relationships of the *E. bieneusi* genotypes identified here and the known ones (GenBank). The numbers on the branches represent % bootstrap values for 1,000 replicates; the values generated >50% are shown beside the nodes. The host origin, accession number, and genotype designation were used to identify individual sequences. The filled squares and circles represent the known and novel genotypes identified here, respectively.

The results of phylogenetic analysis showed that all genotypes were from the two groups (Figure 2). These included 13 genotypes (CHC5, CHG19, CHN4, D, EbpA, EbpC, Henan-II, HNPL-I, HNPL-II, HNPL-III, KIN-1, macaque4, and SHW7) in the zoonotic group 1 and the remaining four genotypes (HNPL-IV, HNPL-V, HNR-VI, and SHR1) in group 12. The group 1 genotypes were classified as follows: subgroup1a (macaque4 and D); subgroup 1b (SHW7); subgroup 1c (CHN4); subgroup 1d (CHC5, CHG19, EbpC, Henan-III, HNPL-I, and HNPL-II); and subgroup 1e (EbpA and HNPL-III).

## Discussion

To the best of our knowledge, only one study, based in four provinces (Hainan, Chongqing, Guangdong, and Jiangxi) of China, has identified the *E. bieneusi* pathogen in masked palm civets globally (12). They had an average infection rate of 53.3%, which is composed of Hainan, Chongqing, Guangdong, and Jiangxi at 86.1, 85.9, 46.2, and 35.2%, respectively (12). The present study has reported a 51% prevalence of *E. bieneusi*, with infection rates ranging from 3.6 to 80.0% among the five farms sampled from Hainan, China. Generally compared with the previous report, our study has established a lower prevalence of *E. bieneusi* in masked palm civets (12). Presumably, the high infection rate could be attributed to insanitation in the two sampled farms in Hainan and Chongqing. Besides, each cage had ~2–6 animals, and there was minimal interaction with neighboring cages. Meanwhile, 73.3% (652/889) of these animals were younger than 2 years (12). Notably, the masked palm civets were kept in groups of two animals (a male and a female) per cage in our study. All these animals were older than 2 years and were assumed to be healthy while sampling. This could be the reason behind the differences in the prevalence of *E. bieneusi* in masked palm civets. Research has shown that this prevalence could be affected by multiple factors, like the sample size, host health, animal practices, and the detection methods. Therefore, it is difficult to determine the actual infection rate of a specific animal species within a particular region.

Previous epidemiological studies have reported 13 *E. bieneusi* genotypes (Peru8, J, PL1 to PL11) in masked palm civets with genotypes Peru8 and J being zoonotic (12). Here, we identified 12 previously known genotypes (CHC5, CHG19, CHN4, D, EbpA, EbpC, Henan-II, HNR-VI, KIN-1, macaque4, SHR1, and SHW7) and five novel genotypes (HNPL-I to HNPL-V). Genotypes HNR-VI and SHR1 showed predominance in the investigated masked palm civets and were detected in 78.9% (101/128) of *E. bieneusi* isolates. Genotype HNR-VI was initially detected in Asiatic brush-tailed porcupine from Hainan, China (8). In contrast, genotype SHR1 was identified in rats and pet snakes from Henan and Beijing of China (15, 16). Thus, these findings have suggested that the two genotypes could be transmitted between rats and civets and that in the future, there is a need for more research to confirm their true host range.

Among the other known *E. bieneusi* genotypes, genotypes D, EbpC, CHN4, EbpA, and Henan-III are known to infect humans (17). Genotypes D, EbpA, and EbpC are the three most common genotypes detected in humans, and they have been isolated in various animal hosts and water samples (2). Genotypes KIN-1, CHN4, and Henan-III have only been discovered in a few human cases of microsporidiosis, with genotype KIN-1 in a Cameroonian, while genotypes CHN4 and Henan-III in three and one Chinese, respectively (18–20). Additionally, they have also been found in some animals from China. For instance, genotype KIN-1 has been found in pigs, cattle, deer, and goats (21–24); genotype CHN4 in calf and coypus (25, 26); and genotype Henan-III in NHPs, pet snakes, pigs, and birds (6, 27–29). The above results indicate that masked palm civets could harbor the different zoonotic genotypes. Hence, the likelihood for veterinary workers, farm management personnel, and other contacts to become infected with *E. bieneusi* is a matter of real concern.

Genotype SHW7 has only been identified in water from Shanghai, China, but its original source is unclear (30). In this study, this genotype was found in civets, suggesting that they could be its hosts. The remaining known genotypes identified in this study (CHC5, CHG19, and macaque4) have only been established in animals, for example, CHC5 in cattle, pigs, and wild boars; CHG19 in pigs, wild boars, goats, and horses; and lastly, macaque4 in the macaque (31–34). Thus, it is imperative to explore whether these genotypes could be present in humans.

This study has identified that among the five novel genotypes, HNPL-I, HNPL-II, and HNPL-III were genetically similar to the human-pathogenic EbpC and EbpD genotypes, and they were categorized into group 1. The other two novel genotypes (HNPL-IV and HNPL-V) have shown a base variation compared with the SHR1 genotype, and they were sorted into group 13. Group 1 was suspected to have zoonotic potential as it had maximum human-pathogenic genotypes and possessed 94% of the known *E. bieneusi* ITS sequences (2).

There are some limitations in sample collection in this study. (1) Only those farms were chosen whose owners agreed to participate in the study and had easy access to animals for sampling. (2) The collected samples accounted for ~30% of the total animals on each farm instead of all animals on each farm. (3) The masked palm civets were categorized into groups of two (a male and a female) per cage, and to avoid duplicate sampling, only one specimen was collected per cage. (4) All animals were aged 2–4 years and were healthy. So the actual infection rate may be underestimated.

## Conclusion

This study has shown that the infection rates of *E. bieneusi* in masked palm civets from Hainan of China are relatively high at 51.0% and provide baseline data to control and prevent microsporidiosis in farm-related communities. In total, we identified 17 genotypes in the masked palm civets; the two genotypes (HNR-VI and SHR1) were considered host-adapted genotypes and likely pose little risk of zoonotic transmission; however, the six known zoonotic genotypes CHN4, D, EbpA, EbpC, Henan-III, and KIN-1 should be considered potential threats to public health. Meanwhile, the identified novel genotypes of *E. bieneusi* here provide new insights into the genotypic variations of this pathogen.

## Data Availability Statement

The datasets presented in this study can be found in online repositories. The names of the repository/repositories and accession number(s) can be found in the article/supplementary material.

## Author Contributions

GL and SL conceived and designed the experiments. G-XR, YQ, JL, JP, and YZ performed the experiments. WZ and G-XR analyzed the data. WZ wrote the paper. FT and HH contributed the reagents, materials, and analysis tools. SL and GL critically revised the manuscript. All authors read and approved the final version of the manuscript.

## Funding

This work was supported by the Major Science and Technology Program of Hainan Province (ZDKJ202003), Research project of Hainan academician innovation platform (YSPTZX202004), Hainan talent development project (SRC200003), the National Natural Science Foundation of China (Nos. 82060375 and 81760378), Innovation Research Team Project of Hainan Natural Science Foundation (2018CXTD340), and the Open Foundation of Key Laboratory of Tropical Translational Medicine of Ministry of Education, Hainan Medical University (2020TTM004).

## Conflict of Interest

The authors declare that the research was conducted in the absence of any commercial or financial relationships that could be construed as a potential conflict of interest.

## Publisher's Note

All claims expressed in this article are solely those of the authors and do not necessarily represent those of their affiliated organizations, or those of the publisher, the editors and the reviewers. Any product that may be evaluated in this article, or claim that may be made by its manufacturer, is not guaranteed or endorsed by the publisher.
